# ICTV Virus Taxonomy Profile: *Ascoviridae*

**DOI:** 10.1099/jgv.0.000677

**Published:** 2017-03-17

**Authors:** Sassan Asgari, Dennis K Bideshi, Yves Bigot, Brian A Federici, Xiao-Wen Cheng, ICTV Report Consortium

**Affiliations:** ^1^​ School of Biological Sciences, The University of Queensland, Brisbane, QLD 4072, Australia; ^2^​ Department of Natural and Mathematical Sciences, California Baptist University, 8432 Magnolia Avenue, Riverside, CA 92504, USA; ^3^​ UMR INRA-CNRS 7247, PRC, Centre INRA de Nouzilly, 37380 Nouzilly, France; ^4^​ Department of Entomology, University of California, Riverside, CA 92521, USA; ^5^​ Interdepartmental Graduate Programs in Microbiology, University of California, Riverside, CA 92521, USA; ^6^​ Department of Microbiology, Miami University, 32 Pearson Hall, Oxford, OH 45056, USA

**Keywords:** *Ascoviridae*, ICTV report, taxonomy

## Abstract

The family *Ascoviridae* includes viruses with circular dsDNA genomes of 100–200 kbp characterized by oblong enveloped virions of 200–400 nm in length. Ascoviruses mainly infect lepidopteran larvae and are mechanically transmitted by parasitoid wasps in which they may also replicate. Most known members belong to the genus *Ascovirus*, except one virus, that of the genus *Toursvirus*, which replicates in both its lepidopteran and parasitoid vector hosts. Ascoviruses cause high mortality among economically important insect pests, thereby controlling insect populations. This is a summary of the current International Committee on Taxonomy of Viruses (ICTV) Report on the taxonomy of the *Ascoviridae*, which is available at www.ictv.global/report/ascoviridae.

## Virion

**Fig. 1.**
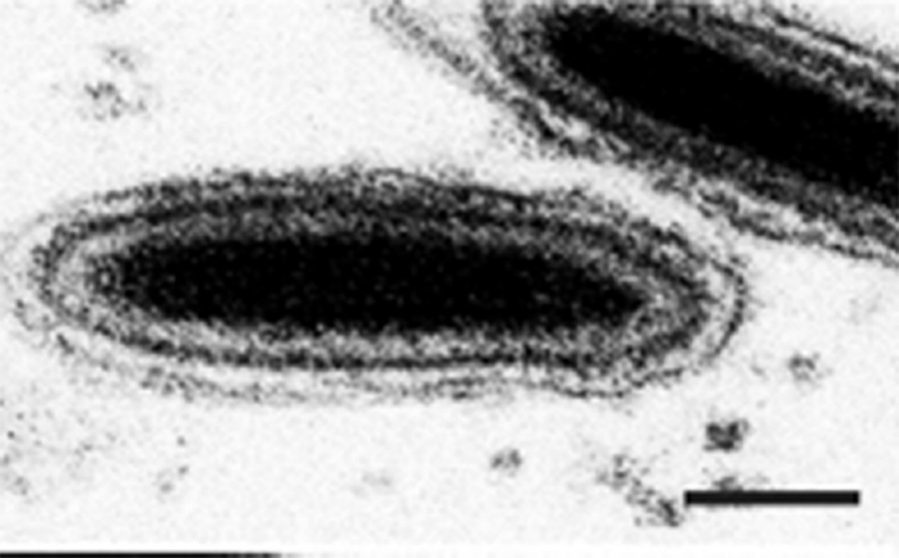
Morphology of ascovirus virions. Ultrathin longitudinal-section through typical ascovirus virions (Spodoptera frugiperda ascovirus 1a). The virion consists of an inner particle and an outer envelope. The inner particle is complex and contains a DNA–protein core surrounded by an apparent unit membrane, the external surface of which bears a layer of distinctive protein subunits. Bar, 50 nm.

Virions of ascoviruses are bacilliform, ovoidal or allantoid in shape, and depending on the species, have complex symmetry and are large, measuring about 130 nm in diameter by 200–400 nm in length (Table 1, Fig. 1; [1]).

**Table 1.** Characteristics of the family *Ascoviridae*

Typical member	Spodoptera frugiperda ascovirus 1a (AM398843), species *Spodoptera frugiperda ascovirus 1a* (AM398843), genus *Ascovirus*
Virion	Enveloped, 130 nm in diameter by 200–400 nm in length, at least 20 polypeptides
Genome	100–200 kbp of circular dsDNA with 117–180 genes
Replication	Nuclear, with cell cleavage into virion-containing vesicles that turn the host haemolymph milky white
Translation	From transcribed mRNAs
Host range	Lepidopteran insect larvae, mostly members of the family Noctuidae
Taxonomy	Two genera *Ascovirus* and *Toursvirus*

## Genome

The genome consists of a single molecule of circular dsDNA ranging in size from 100 to 200 kbp. Ascovirus genomes contain from 117 to 180 genes, of which 40 are common among them. While the genome organization of members of the *Ascovirus* is collinear, that of the member of *Toursvirus* is quite different. Based on phylogenetic analyses, it appears that ascoviruses emerged recently from an invertebrate ancestral iridovirus lineage [2].

## Replication

Ascoviruses initiate replication in the nucleus of infected cells. The nucleus enlarges and ruptures followed by cleavage of the cell into a cluster of virion-containing vesicles, a characteristic typical of all known viruses of this family (Greek *asco*=sac) [3]. Virion assembly becomes apparent after the nucleus ruptures. The first recognizable structural component of the virion to form is the multilaminar layer of the inner particle. Based on its ultrastructure, this layer consists of a unit membrane and an exterior layer of protein subunits. As the multilaminar layer forms, the dense DNA–protein core assembles along the inner surface. After the inner particle is assembled, it is enveloped by a membrane that is synthesized *de novo,* or elaborated from cell membranes, within the cell or vesicle. In members of *Spodoptera frugiperda* *ascovirus 1a*, the type species of the genus *Ascovirus*, virions are occluded in an occlusion body composed of mini vesicles and protein.

## Taxonomy

### Ascovirus

This genus includes three species whose members infect various members of the insect family Noctuidae, many species of which are economically important. Ascoviruses are difficult to transmit orally, and experimental studies as well as field observations indicate that virions are transmitted horizontally by endoparasitic wasps of the families Braconidae and Ichneumonidae (Hymenoptera). During egg laying, the ovipositor of female wasps becomes contaminated with virions circulating in the haemolymph of infected caterpillars. Wasps contaminated in this manner subsequently transmit ascovirus virions to new caterpillar hosts during oviposition [4].

### Toursvirus

This genus includes only one species whose members are confined to the lepidopteran family Yponomeutidae, in which they replicate extensively. Virus of this species also replicates in its ichneumonid vector, *Diadromus pulchellus*, but replication is limited and relatively few virions are produced in comparison to the number generated in the lepidopteran host [5]. In the wasp, the virus is transmitted vertically when the viral genome is carried as unintegrated DNA in the nuclei of infected cells.

## Resources

Full ICTV Online (10th) Report: www.ictv.global/report/ascoviridae.
